# Anti‐Metastatic and Anti‐Angiogenic Activities of Core–Shell SiO_2_@LDH Loaded with Etoposide in Non‐Small Cell Lung Cancer

**DOI:** 10.1002/advs.201600229

**Published:** 2016-10-08

**Authors:** Yanjing Zhu, Rongrong Zhu, Mei Wang, Bin Wu, Xiaolie He, Yechang Qian, Shilong Wang

**Affiliations:** ^1^ Research Center for Translational Medicine at East HospitalSchool of Life Science and TechnologyTongji UniversityShanghai200092China; ^2^ Department of Respiratory DiseaseBaoshan District Hospital of Integrated Traditional Chinese and Western MedicineShanghai201900China

**Keywords:** anti‐angiogenic, anti‐metastasis, etoposide, lung cancer, SiO_2_@LDH

## Abstract

Currently, nanoparticles have gained a great attention in the anti‐tumor research area. However, to date, studies on the anti‐metastasis action of core–shell SiO_2_@LDH (LDH: layered double hydroxide) nanoparticles remain untouched. Two emerging aspects considered are establishing research on the controlling delivery effect of SiO_2_@LDH combined with anti‐cancer medicine from a new perspective. The fine properties synthetic SiO_2_@LDH‐VP16 (VP16: etoposide) are practiced to exhibit the nanoparticle's suppression on migration and invasion of non‐small cell lung cancer (NSCLC). Both in vitro and in vivo inspection shows that SiO_2_@LDH can help VP16 better function as an anti‐metastasis agent. On the other hand, anti‐angiogenic efficiency, co‐localization, as well as western blot are investigated to explain the possible mechanism. A clear mergence of SiO_2_@LDH‐VP16 and cytomembrane/microtubule may be observed from co‐location images. Results offer evidence that SiO_2_@LDH‐VP16 plays positions on cytomembrane and microtubules. It efficiently inhibits metastasis on NSCLC by reducing vascularization, and eliciting depression of the PI3K‐AKT and FAK‐Paxillin signaling pathways. SiO_2_@LDH‐VP16, the overall particle morphology, and function on anti‐metastasis and anti‐angiogenic may be tuned to give new opportunities for novel strategies for cancer therapy.

## 1 Introduction

Cancer metastasis, rather than the primary tumor, is the cause for most cancer‐related deaths.^1^ In this procedure, tumor cells migrate from the original site to other organs. Before cancer has metastasized, the patient often can be cured by surgery or radiotherapeutics. However, once the tumor cells have spread, the situation becomes more difficult to manage. Even after seemingly effective therapy, metastases may be detected at a later time, even years after primary treatment.^2^ The progress of metastasis remains unpredictable due to its “hidden” sheer complexity. Consequently, current curative effects are not optimistic.^3^, ^4^ Successful suppression of metastasis is plagued by a lack of safe and effective medicines, as well as technique. Efficient approaches for addressing cancer metastasis are urgently needed.

Based on earlier findings, nanoparticle application may be considered as a potential method in anti‐metastasis research.^5^ Nanoparticles have captured much attention, for example, their use as gene transporters, drug carriers, radioactive tracers, and therapies in medicine and other relevant fields.^6^, ^7^, ^8^ Amongst the known existing approaches, nanoparticles have prominent advantages in biosafety, targeting, and releasing control.^9^, ^10^

Layered double hydroxides (LDHs), known as anionic clays, can be expressed with the general formula [M^II^_1‐x_M^III^x (OH)_2_]^x+^(A^n−^)_x/n_• mH_2_O, where typically, M represents the metal and A is the interlayer anion.^11^ LDH has been demonstrated as a promising carrier for drug delivery, according to its expandable interlayer space, low cytotoxicity, and high biocompatibility.^12^, ^13^, ^14^, ^15^ Our team's previous research has already confirmed that layered double hydroxide loaded with etoposide (LDH‐VP16) is very effective in inhibiting A549 cell migration and invasion in vitro via the mTOR/AKT and STAT pathways.^16^ Human pulmonary adenocarcinoma (A549) cells were widely used as a model of non‐small cell lung cancer (NSCLC).^17^ Because of its poor prognosis, lung cancer is one of the deadliest types of cancer.^18^, ^19^ NSCLC has a high probability of metastasis, accounting for approximately 85% of lung cancer cases.^20^ Since metastasis is inherently troublesome to explore, the further study requires an in vivo pulmonary model. In further research of introducing LDH into an anti‐metastasis area, we continue to choose VP16 as a model drug. VP16 is a topoisomerase inhibitor, which has been generally used for the treatment of various cancers.^21^ Previous works also prove that LDH can strengthen the anti‐tumor effect of VP16 by substantially reducing its cytotoxicity and enhancing its poor bioavailability.^22^

However, traditional LDH exhibits imperfect morphology due to serious aggregated powders by Bernal stacking, which would limit the far‐sighted development of practical applications.^23^ Our recent findings propose that well‐dispersed 3D SiO_2_@LDH hierarchical spheres serve as an immune adjuvant.^24^ Such core–shell LDH materials were constructed using layer‐by‐layer deposition and were shown to be an excellent non‐viral gene delivery system, according to our exploration. Heretofore, research of SiO_2_@LDH remains in synthesis and characterization, however, our research concentrates on expanding beyond this.

This study focuses on exploring the potential and mechanism of SiO_2_@LDH as a drug carrier in anti‐metastases, both in vitro and in vivo. Compared to previous research, our work moves core–shell SiO_2_@LDH on to a more practical level, since it may be incorporated into mature animal models, clinical medicine, and cancer treatments.

## 2 Results

### 2.1 Physicochemical Characterization of SiO_2_@LDH‐VP16

**Figure 1:**
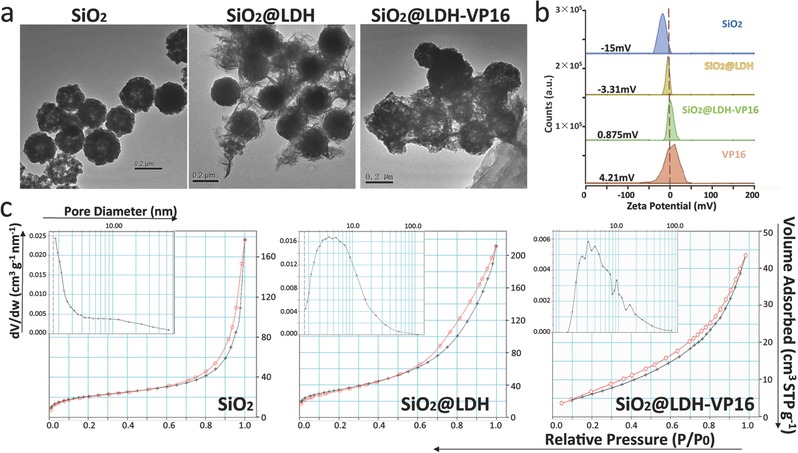
a) TEM images of SiO_2_, SiO_2_@LDH, and SiO_2_@LDH‐VP16. b) Zeta potential distribution for SiO_2_, SiO_2_@LDH, SiO_2_@LDH‐VP16, and VP16. c) N_2_ adsorption/desorption isotherms and BJH pore‐size distribution curve obtained from the adsorption branch of SiO_2_, SiO_2_@LDH, and SiO_2_@LDH‐VP16.

According to transmission electron microscope (TEM) observations (**Figure** **1**a), SiO_2_ nanoparticles had an average diameter of 200 nm with a clear mesoporous structure. SiO_2_@LDH has a 20 nm extend with lamellar crystallized LDH on the surface, whereas SiO_2_@LDH‐VP16 showed a denser dimensional core–shell architecture compare to SiO_2_ nanoparticles alone.^24^ Result of dynamic light scattering (DLS) of SiO_2_@LDH‐VP16 showed an average diameter of about 217 nm, which supported the observation of TEM photo (Figure S3, Supporting Information). The PDI value is 0.272, which indicated a moderately polydisperse distribution type of the nanoparticles, where the distribution is neither extremely polydisperse, nor in any sense narrow or broad. Besides, Figure S1 in the Supporting Information showed that SiO_2_@LDH‐VP16 could be suspended well in phosphate‐buffered saline (PBS) compared to VP16 alone. SiO_2_@LDH‐VP16 has better quality of dispersion, and according to the animal study, it is suitable for in vivo injection to the mice.

A zetasize measured the surface charge of both nanoparticles and free drug. As shown in Figure 1b, the zeta potential of SiO_2_ was ‐15 ± 1.2 mV and SiO_2_@LDH was ‐3.31 ± 0.2 mV. In this case, SiO_2_@LDH revealed properties more closely related to electrical neutrality, and can reduce nonspecific impurities in combination in vivo and can raise the delivery efficiency. The zeta potential value of VP16 was 4.21 ± 0.3 mV, in addition, it can combine with the slightly electronegative SiO_2_@LDH. SiO_2_@LDH‐VP16 was 0.875 ± 0.01 mV and it affirms that the drug is well loaded.

As shown in Figure 1c, the Brunauer–Emmett–Teller (BET) adsorption–desorption isotherms of SiO_2_, SiO_2_@LDH can be categorized as type‐IV hysteresis, which can reflect a mesoporous structure.^25^ However, no significant similar mesoporous‐ structure curve was found in the BET result of SiO_2_@LDH‐VP16. The Barrett–Joyner–Halenda pore size distribution curves are also showed in Figure 1c, all of the three samples appeared a pore size range below 50 nm, and as listed in **Table** **1**, the mean diameter of SiO_2_, SiO_2_@LDH, and SiO_2_@LDH‐VP16 was 14.27, 10.73, and 3.11 nm, respectively. This decreased change proved that the drug was well loaded in SiO_2_@LDH. The calculations derived from the adsorption branch show that there is no peak value observed for SiO_2_, which indicates that the size of SiO_2_ is determined randomly. In comparison, the SiO_2_@LDH showed a centralized distribution, which corresponds to the hollow inner structure of SiO_2_@LDH. The BET results demonstrated that SiO_2_@LDH has an excellent thermal stability compared to the unmodified SiO_2_.^26^, ^27^ Besides, based on Table 1, the specific surface area of each nanoparticle was 76.82, 122.06, and 25.65 m^2^ g^−1^, respectively, which suggests the better loading capacity of SiO_2_@LDH. The noticeable decrease in pore size of SiO_2_@LDH‐VP16 was considered due to the existence of drug. According to the measurement, the drug‐loading rate of SiO_2_@LDH was about 39%, while SiO_2_ and LDH were 17% and 25%, respectively.

**Table 1:** N_2_ adsorption/desorption measurement results of SiO_2_, SiO_2_@LDH, and SiO_2_@LDH‐VP16, respectively

Electrode	S_BET_ [m^2^ g^−1^]	D [nm]	*V_t_* [cm^3^ g^−1^]
SiO_2_	76.82	14.27	0.2740
SiO_2_@LDH	122.06	10.73	0.3273
SiO_2_@LDH‐VP16	25.65	3.11	0.0673

### 2.2 Dose‐Dependent Cytotoxicity of SiO_2_@LDH‐VP16 on A549 Apoptosis

**Figure 2:**
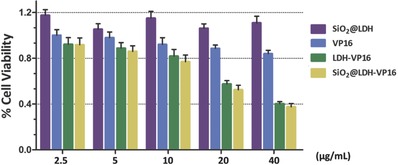
MTT analysis of A549 cell viability treated with VP16, LDH‐VP16, SiO_2_@LDH, and SiO_2_@LDH‐VP16 after 24 h. Error bar represented means of three independent experiments.

The biological safety of SiO_2_@LDH‐VP16 was evaluated by MTT assays and the results are shown in **Figure** **2**. According to the histogram, over 90% of cells survived after being treated with SiO_2_@LDH‐VP16 for 24 h at a concentration of 2.5 and 5 μg mL^−1^. However, it showed certain cytotoxicity when the concentration reached 20 μg mL^−1^, as it may cause cell apoptosis.

### 2.3 Enhanced Inhibition of SiO_2_@LDH‐VP16 Against Lung Cancer Metastasis In Vitro and In Vivo

**Figure 3:**
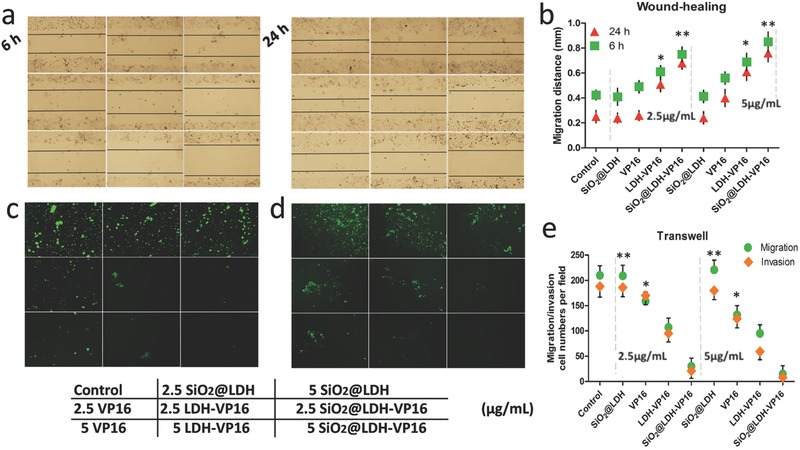
Enhanced anti‐metastasis effect of SiO_2_@LDH‐VP16 on A549 cell. Wound‐healing assays a) were performed to assess cell migration. Cells were treated with 2.5 and 5 μg mL^−1^ of VP16, LDH‐VP16, SiO_2_@LDH, and SiO_2_@LDH‐VP16 for 6 and 24 h or untreated. Representative images of treated and untreated cells are shown (40× magnification). Distance migrated by cells b) at different time points are shown. Migration c) and invasion chamber assays d) were utilized to show the effect of SiO_2_@LDH‐VP16 in vitro. Cells were treated with the same concentration as the wound‐healing assay for 24 h or untreated. Representative images of treated and untreated cells are shown (200× magnification). Number of migrating and invading cellse) were counted. The values represent the mean ± SD of three independent experiments (**P < 0.01).

The ability of SiO_2_@LDH‐VP16 to suppress migration and invasion in vitro was investigated using wound healing and transwell migration assay. As shown in **Figure** **3**a, the SiO_2_@LDH‐VP16 group had a narrower migration distance and fewer migratory cells compared to free VP16 and LDH‐VP16, while the SiO_2_@LDH group had a negligible difference from the control in both aspects. Transwell invasion results also matched.

**Figure 4:**
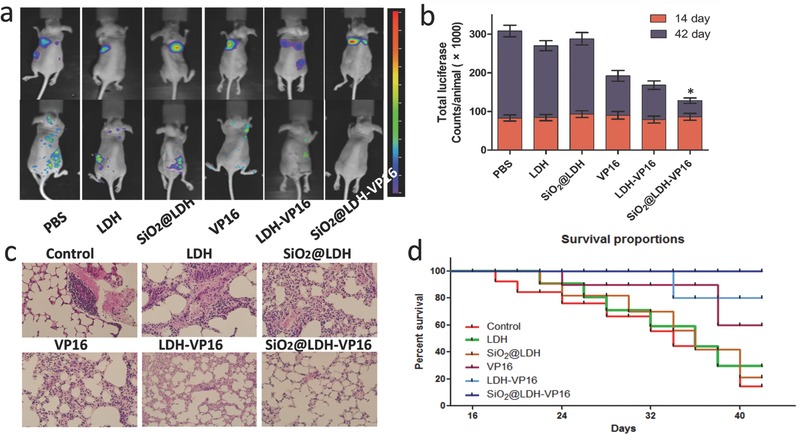
SiO_2_@LDH‐VP16 prevents the establishment of NSCLC metastasis. In vivo total‐body bioluminescence images a) of athymic nude mice in dorsal and ventral positions (IVIS@Imaging System) injected i.v. with A549‐Luc cells. Quantitative analysis of metastasis b) (estimated by total luciferase counts per animal) in athymic nude mice. Representative images of histological analysis c) (H&E staining) of lung micrometastasis. Each of the six groups is denoted by survival percent d): the survival of SiO_2_@LDH‐VP16 group is significantly different from the other treated groups (*p* = 0.05) and from the control group (*p* < 0.01).

The metastatic mouse tumor model was chosen to explore the possible anti‐cancer effect of SiO_2_@LDH‐VP16.^28^ The effect on anti‐metastasis of SiO_2_@LDH‐VP16, in vivo study was carried out using the tail vein pulmonary model, as shown in **Figure** **4**. Bioluminescence imaging showed that the mice in the SiO_2_@LDH‐VP16 group formed relatively weak metastasis, compared to PBS group. In keeping with the in vitro outcome, SiO_2_@LDH‐VP16 had a more potent inhibitory action than LDH‐VP16, while neither LDH nor SiO_2_@LDH had a decided effect. Histological examination gave proof of the presence of pulmonary micrometastasis in the control and blank nanoparticles group. The VP16 and the LDH‐VP16 group showed less inflammatory cell infiltration than the control. A visualized enhanced efficiency can be observed in the SiO_2_@LDH‐VP16 treatment group. In addition, SiO_2_@LDH may significantly increase the survivability of mice injected with tumor cells, as all the mice survived compared to other groups that had a measurable rate of death during the experiment.

### 2.4 Anti‐Angiogenic Efficiency of SiO_2_@LDH‐VP16

**Figure 5:**
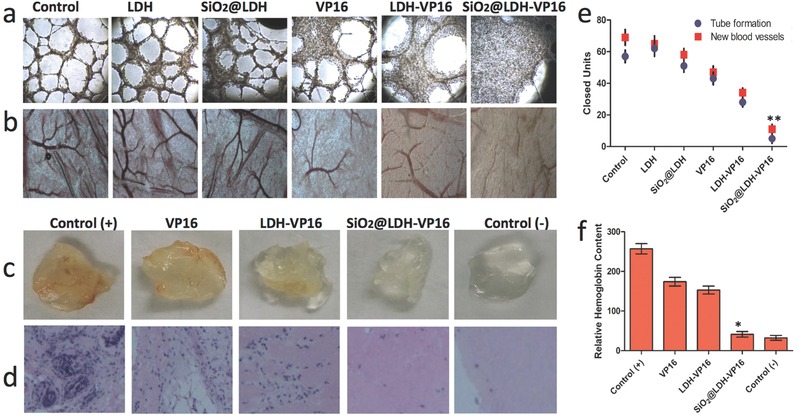
SiO_2_@LDH‐VP16 inhibits angiogenesis. a) Tube formation was photographed; HUVECs were treated with 5 μg mL^−1^ VP16, LDH, SiO_2_@LDH, LDH‐VP16, or SiO_2_@LDH‐VP16 for 24 h. The same concentration nanoparticles or medicines were implanted in b) chicken chorioallantoic membrane (CAM) and neovascularization was photographed. The numbers indicate percentage of tube‐like structures and the numbers indicate percentage of new blood vessels e) arising from the existing blood vessels in naïve CAM. c) Macroscopic analysis of matrigels from one representative experiment. Vessel formation was assessed after injection of mice with matrigel plugs containing VEGF alone (positive control) or in combination with 5 μg mL^−1^ VP16, LDH‐VP16 or SiO_2_@LDH‐VP16 for 10 d. d) All the plugs were fixed with formalin and embedded in paraffin. Matrigel plug sections were stained with H&E to show infiltrating cells. f) Infiltrating cells into the matrigel plugs were quantified as cells per section. *n* = 3–6 matrigel plugs, *p < 0.05.

As shown in **Figure** **5**, compared to free drug or LDH‐VP16, in tube formation assay, SiO_2_@LDH‐VP16 showed a more prominent inhibition efficiency of angiogenesis, where almost no tube‐like structure can be observed. As to the chorioallantoic membrane (CAM) assay, neovascularization was significantly suppressed when treated with SiO_2_@LDH‐VP16, in accordance with the lack of tube formation. Analysis of in vivo matrigel plugs also confirmed that SiO_2_@LDH‐VP16 reduced the degree of vascularization. The hematoxylin and eosin (H&E) photos further prove the above results that SiO_2_@LDH‐VP16 has a marked decreased infiltration, similar to that observed in the negative control.

### 2.5 SiO_2_@LDH Increase Cellular Uptake of VP16 in A549 Cells

**Figure 6:**
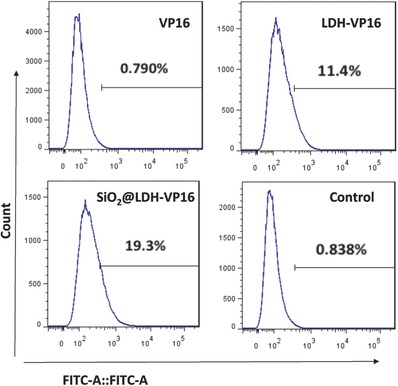
SiO_2_@LDH enhanced cellular uptakes of VP16. A549 cell was treated with 5 μg mL^−1^ VP16, LDH‐VP16, or SiO_2_@LDH‐VP16 (stained with FITC) for 24 h and analyzed by FACS analysis.

To see more details of how SiO_2_@LDH‐VP16 enters the cells and functions intracellularly, cellular uptake was employed. The cellular uptake of SiO_2_@LDH‐VP16 is assessed by fluorescence‐activated cell sorting (FACS) analysis, as evident in **Figure** **6**. After 24 h treatment of SiO_2_@LDH‐VP16, 19.3% of the A549 group displayed a positive stain. LDH‐VP16 also showed a promising effect, with a positive value of 11.4% whereas the cells cultured with VP16 only had 0.79%.

### 2.6 Co‐Localization of SiO_2_@LDH in A549 Membrane and Microtubule

**Figure 7:**
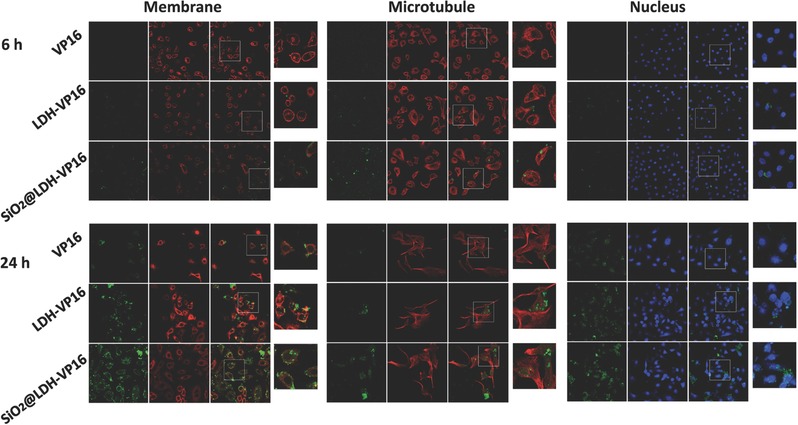
Co‐localization of SiO_2_@LDH in A549. A549 cells were incubated with 10 μg mL^−1^ VP16, LDH‐VP16, or SiO_2_@LDH‐VP16 (stained with FITC). After 24 h, cells were stained with Dil, Tubulin, or DAPI to show membrane, microtubule or nucleus, respectively. Fluorescence micrographs were gain by the confocal microscope (Leica TCS SP5).

The confocal assay was utilized to explore the cellular uptake of SiO_2_@LDH‐VP16. **Figure** **7** clearly verified the result of FACS, since both LDH‐VP16 and SiO_2_@LDH‐VP16 revealed an increase of fluorescein isothiocyanate (FITC) fluorescence intensity, besides, SiO_2_@LDH, which had a notably improved efficacy of raising the uptake of VP16 in A549 cell. Further analysis showed that most of the SiO_2_@LDH‐VP16 accumulations overlapped with the cell membrane and microtubule. However, in the nucleus, only some inconspicuous signal may be observed.

### 2.7 SiO_2_@LDH‐VP16 Regulate the PI3K‐AKT and FAK‐Paxillin Signaling Pathways

**Figure 8:**
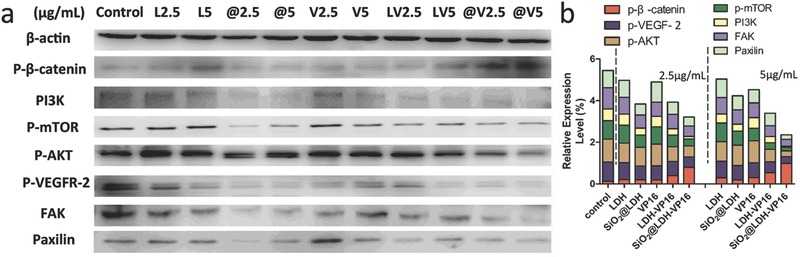
Effects of SiO_2_@LDH‐VP16 on certain signaling pathways in A549 cells. a) Western blot analysis of the p‐ß‐catenin, PI3K, p‐mTOR, p‐AKT, p‐VEGFR‐2, FAK, and Paxillin protein levels from A549 cells treated with concentration at 2.5 or 5 μg mL^−1^ of VP16, LDH, SiO_2_@LDH, LDH‐VP16, or SiO_2_@LDH‐VP16. b) Quantification of the above‐mentioned several protein levels normalized to the internal control. (*n* = 3 per group and mean ± SD).

Additionally, to better understanding the mechanism, a western blot assay was performed. As evident in **Figure** **8**a, SiO_2_@LDH‐VP16 remarkably weakened the band value of phospho‐VEGFR‐2, PI3K, phospho‐mTOR, and phospho‐AKT, whereas the phosph‐ß‐catenin signal was significant increased after SiO_2_@LDH‐VP16 treatment. The mean gray degree of FAK and Paxillin was decreased as well. ß‐actin was used as an internal reference. The total gray value of every group is shown in the histogram (Figure 8b).

## 3 Discussion

Metastasis has become the most threatening part of the oncogenic process. The in vitro anti‐tumor action of LDH‐VP16 has already been proved as mentioned above. However, to achieve a more reliable effect, limitations of traditional LDH have to be overcome. Traditional LDH has an aggregation problem caused by Bernal stacking, and the simple layer structure leads limited drug loading ratio (25%). Specific surface area (SSA) is an important property of porous solid materials, which measured as the total surface area of a material per unit of mass.^29^ Previous researches have made clear that higher surface areas can contribute to improve loading capacity of nanoparticles.^30^, ^31^ Results show the increased SSA of SiO_2_@LDH compared with SiO_2_ (76.82–122.06 m^2^ g^−1^). Besides, in Figure S2 in the Supporting Information, the FTIR spectra of free VP16, SiO_2_@LDH, and SiO_2_@LDH‐VP16 were shown. Free VP16 showed the following selected bands: 2923 cm^−1^ (C—H stretch), 1770 cm^−1^ (C=O stretch of ester bond), 1610 cm^−1^ (C=O stretch of carboxyl methyl), 1110 cm^−1^ (C—O—C stretch), and 1487 cm^−1^ (C=C stretching in the backbone of the aromatic phenyl ring). The spectra of SiO_2_@LDH showed characteristic peaks: 3461.69 and 1384.12 cm^−1^ (—OH stretching vibration and NO^3−^) and 989.41 cm^−1^ (Si—OH bending vibration). The FTIR results suggested that VP16 was well encapsulated by SiO_2_@LDH, since the entire characteristic peaks for free VP16 were also identified in the SiO_2_@LDH‐VP16.

A significant pH sensitive release pattern of the entrapped VP16 from the SiO_2_@LDH‐VP16 formulation was observed in Figure S4 in the Supporting Information. Only about 10% of VP16 released from SiO_2_@LDH at 48 h when the pH value is 7.4. Meanwhile, under pH 5.8, SiO_2_@LDH‐VP16 possessed a sustained drug release over a period of 48 h. The results seem to prove that SiO_2_@LDH has the same pH‐sensitive characteristics as LDH which could be helpful to largely diminish the hematotoxicity.^22^ In first 8 h, an initial fast drug release could be observed due to those drugs located on the surface of SiO_2_@LDH, and then the slow sustained release could be attributed to the diffusion of drug molecules from nanoparticle. The first burst release can contributes to improve the penetration of a drug, while sustained release benefits the irritation effects at high concentrations.

The combination of LDH and SiO_2_ creates a novel nanomaterial with high loading capacity (39%). In the case of this study, since the VP16 loading ratio of SiO_2_ is only 17%, to achieve the best loading ratio, LDH‐VP16 and SiO_2_@LDH‐VP16 were served as potential agent of delivery VP16, and further exploration of their comparison and difference prove that SiO_2_@LDH‐VP16 has the advantage over LDH‐VP16 in anti‐metastatic efficiency.

Emerging two aspects, we evaluate both the in vitro and in vivo anti‐metastatic activity of the novel 3D core–shell SiO_2_@LDH loaded with VP16. The result of TEM, zeta potential, and BET demonstrated the successful loading of VP16 into the SiO_2_@LDH. SiO_2_@LDH may be considered an ideal carrier for the drug due to its extremely sizable areas. The cell experiments have confirmed that the drug‐delivery hybrid system strongly promotes the efficiency of VP16 in inhibiting A549 cell migration and invasion. In accordance with the in vitro results, fluorescence living imaging proves the distinct enhanced anti‐metastasis impact of SiO_2_@LDH‐VP16. In H&E staining, lung sections of SiO_2_@LDH‐VP16 reveal less nuclear matter and highly disseminated cytoplasmic structures compared to other groups. MTT and survival curve (Figure 4d) clearly prove the biosafety of SiO_2_@LDH and SiO_2_@LDH‐VP16 only has a negligible impact on cell apoptosis at the designated dose, therefore, it would not have significant influence in evaluating cancer cell migration and invasion. To eliminate the effects of cell apoptosis on the assessment of anti‐metastasis efficiency, we chose 2.5 and 5 μg mL^−1^ as the suitable concentration for further migration investigation.^32^

In the long run, the only evidence of efficiency is inadequate. The mechanism of the anti‐metastasis effect of SiO_2_@LDH must be further explained in depth. Tube formation, CAM, and matrigel plugs indicate reduced angiogenesis, which is a crucial progress in cancer metastasis.^33^ Metastasis occurs when tumor cells from a primary organ migrate to another site, where they form a new tumor. A new blood supply may be developed to support the metabolism of the tumor. The formation of those vessels around the cancer site is called angiogenesis.^34^ Angiogenesis is a cascade of procedures emanating from microvascular endothelial cells. Cancer cells can escape from a tumor site and enter the blood circulation system via these new blood vessels or through the lymphatic system.^35^ Once arrest the new site, the continuous growth of the cancer cells must be sustained by the new vessels, thus, a micrometastasis may continue to form a macroscopic tumor.^36^, ^37^, ^38^, ^39^ Therefore, suppression of angiogenesis can contribute to anti‐metastasis. The considerable decrease of blood vessels in the SiO_2_@LDH‐VP16 group illustrates the anti‐angiogenesis effect of the drug‐delivery hybrid system. Consequently, SiO_2_@LDH‐VP16 can inhibit expansion of vascularized tumors, both locally and metastasized.

To see more details of how SiO_2_@LDH‐VP16 being taken in and function, cellular uptake was employed. A clear mergence of SiO_2_@LDH‐VP16 and cytomembrane can be observed from co‐location images. Cancer metastasis processes are related to membrane variation. The “Membrane flow model” is one of the possible mechanisms describing cell migration and invasion. Stretching of the membrane has a great impact on cell endocytic progress.^40^, ^41^ Integrins are transported through endocytosis as well and metastasis‐related receptors such as vascular endothelial growth factor (VEGF) are distributed in the membrane. Besides, the degree of fluorescence of FITC, which merged with Tubulin‐Tracker Red is apparent but not as strong as Dil. Microtubule activity is crucial for cell division and other key events. Disrupting microtubule action may contribute to inhibiting tumor growth and angiogenesis. Targeting microtubule to suppress the dynamics has become a very important field in the current anti‐cancer drug industry.^42^, ^43^, ^44^ The co‐location photos indicate that SiO_2_@LDH‐VP16 is acting on the cytomembrane and microtubules to suppress the cancer metastasis. Besides, our previous publications have already done some exploration on the mechanism of uptake of VP16 and SiO_2_@LDH, results illuminated that the way LDH‐VP16 and SiO_2_@LDH get into cells depends on the caveolae‐mediated pathway.^16^, ^24^ The standard of categorizing pathways depends on the production of distinct derivatives. Moreover, the cellular uptake progress ought to be energy‐dependent.^24^, ^45^ The clear perception of cellular uptake matters to further study on the mechanism.

Additionally, for better understanding the mechanism, western blot assay was performed. The findings argue that the activated receptor such as VEGF plays a role as an antigenic factor and that VEGFR‐2 is necessary for tumor metastasis.^46^ Western blot bands demonstrated a decrease in phosphorylation of VEGFR‐2 after treatment with SiO_2_@LDH‐VP16 on A549 cells. There have been studies showing that suppression the phosphorylation of VEGFR‐2 led to inhibition of cancer cell migration and metastasis.^47^, ^48^ The prosurvival activity of phosphoinositide 3 kinase (PI3K)/Akt pathway has been identified that actively engages with the migratory process in motile cells, including metastatic cancer cells. Cancer cell migration and invasion malignant progression attenuates by interfering the PI3K/AKT‐media cell motility impairs development.^49^ mTOR and ß‐catenin are both classic downstream of PI3K/AKT pathway.^50^, ^51^ FAK and paxillin, crucial components in integrin‐regulated signaling, facilitate to both cell matrix and adhesions and establish roles as positive regulators of cell migration.^52^ Given the above, accumulating evidence suggests that PI3K‐AKT and FAK‐Paxillin are both well‐established classic intracellular pathways associated with cancer metastasis.^53^, ^54^ An enhanced weakened signal in these two pathways explains the possible molecular mechanism of SiO_2_@LDH‐VP16 on the superiority of anti‐metastasis.

## 4 Conclusion

**Figure 9:**
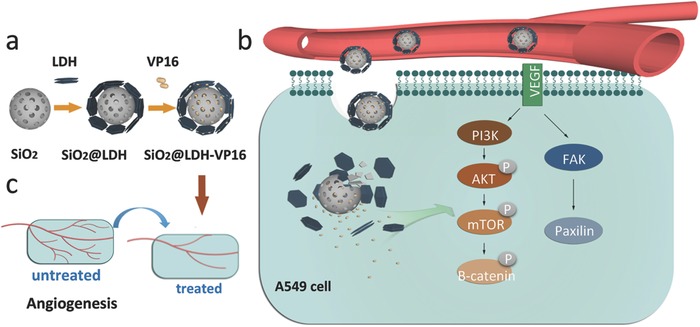
Schematic diagram illustrates and the mechanism b) underlying the inhibition of A549 cells by SiO_2_@LDH‐VP16. c) SiO_2_@LDH‐VP16 has effect of anti‐angiogenesis c) and can subsequently inhibit the PI3K‐AKT and FAK‐Paxillin pathway. a) The brief progress of synthesis is also introduced.

In this study, biodegradable SiO_2_@LDH was considered as a promising nanocarrier for clinical drugs, which has defects of low water solubility and intolerable side effects. We explored not only the physical and chemical properties, but also the in vitro and in vivo anti‐metastasis function of SiO_2_@LDH‐VP16. Results show that SiO_2_@LDH‐VP16 has a decent drug loading capability, which efficiently inhibited the cell migration and invasion on A549 cell and reduced metastasis in mice models. Better still, SiO_2_@LDH‐VP16 shows a significant effect in elevating the mice survival rates. The possible mechanism is shown in **Figure** **9**, we find that this impact may be caused by the anti‐angiogenesis action and inhibition of the PI3K‐AKT and FAK‐Paxillin pathway. To sum up, this work provides a fire‐new perspective of using SiO_2_@LDH nanoparticles combined with anti‐cancer medicine. The very suitable features of SiO_2_@LDH nanoparticles make its application in clinical practice can be envisioned.

## 5 Experimental Section

*Nanoparticles Preparation—SiO_2_@LDH Preparation*: The process of synthesis consists of three main steps: preparation of mesoporous silica nanoparticles (SiO_2_), the formation of SiO_2_@AlOOH, addition a coating of LDH nanoplatelets on the surface of SiO_2_@AlOOH.^55^

Briefly, to prepare SiO_2_ particles, hexdecyl trimethyl ammonium bromide (26.7 g, CTAB, 2.5% wt) was preheated to 60 °C trolamine (96 mmol, TEA) and ethysillicate (9 mmol, TEOS) were added into polypropylene (125 mL) and the mixture was heated for 20 min at 90 °C. Then preheated CTAB was added immediately and the final solution was stirred with 600 rpm overnight. For the second progress, Al(OPr)_3_ (11.3 g) was added into deionized water (100 mL) for 20 min stirring at 85 °C. 1.0 m HNO_3_ was used to adjust the pH of Al(OPr)_3_ solution from 3 to 4. The mixture was stirred at 85 °C for 2 h. The solid boehmite (AlOOH) can be formed after water evaporation and re‐dissolved in deionized water (5.8 g, 107 mL) with stirring at 85 °C for 1 h. Then, HNO_3_ (9.5 mL, 1.0 m) was dropped into the AlOOH solution slowly. The solution was refluxed with stirring for another 6 h and then cooling down to room temperature slowly to form the primer sol. Afterward, SiO_2_ particles were dispersed in primer sol and the mix was washed with ethanol. The SiO_2_@AlOOH nanoparticles were dried in air for half an hour. The progress of dispersion, withdrawing, and drying was repeated six times. The last part is to add LDH to the surface of SiO_2_@AlOOH. Mg(NO_3_)_2_•6H_2_O (1.25 g) and NH_4_NO_3_ (2.4 g) were put together in deionized water (80 mL). SiO_2_@AlOOH nanoparticles (0.04 g) were added into above solution for incubated at 80 °C for 24 h. Subsequently, SiO_2_@MgAl‐LDH was rinsed with ethanol for once. The nanoparticles were preserved in water at 4 °C.

*Nanoparticles Preparation—SiO_2_@LDH‐VP16 and LDH‐VP16 Preparation*: To gain SiO_2_@LDH‐VP16, VP16 (129 mg) was dissolved in dimethyl sulfoxide (1 mL, DMSO) and then added into SiO_2_@LDH (18 mL) for stirring overnight at room temperature. Then, the SiO_2_@LDH‐ VP16 was collected using centrifugation at 13 000 rpm. The mixture was dissolved in water (5 mL) and freeze‐dried for 48 h.

Preparation of LDH‐VP16 was implemented following the previous protocol.^16^, ^22^

*Characterizations—Transmission Electron Microscope (TEM)*: The morphological characterizations of SiO_2_, SiO_2_@LDH, and SiO_2_@LDH‐VP16 were established by transmission electron microscope (JEOL, Tokyo, Japan). Each kind of samples was placed on a carbon‐coated copper grid and then observed at 200 kV.

*Characterizations—Zeta Potential*: Zeta potential values of SiO_2_, SiO_2_@LDH, and SiO_2_@LDH‐VP16 were determined at 25 °C by photon correlation spectroscopy (Malvern Zeta‐sizer Nano ZS, Malvern Instrument, UK). Particles size was performed in triplicates following the dilution (100 μL diluted to 1 mL) of the aqueous solution for observing.

*Characterizations—Brunauer–Emmett–Teller Measurements (BET)*: To perform the nitrogen sorption–isotherm measurements, samples were outgassed at 300 °C overnight and measured at 77 K using a Tristar 3000 volumetric adsorption analyzer (Micromeritics). The BET surface area was calculated from the desorption branches in the relative pressure range of 0.05–0.35, and the total pore volume and average pore diameter were evaluated at a relative pressure of about 0.99.

*Characterizations—Drug Loading Ratio Measurements*: To determinate the drug loading efficiency of VP16 in SiO_2_@LDH‐VP16, LDH‐VP16, and SiO_2_‐VP16, the UV–vis spectrophotometer was used. A standard weight (3 mg) of SiO_2_@LDH‐VP16 sample was dissolved by adding a specific amount of ethanol (5 mL). After the sample was completely dissolved, the concentration of the VP16 was determined at a wavelength of 285 nm and calculated according to an already‐obtained calibrating curve (absorbance = 0.00754[VP16]–0.00873, r2 = 0.99991). The drug loading efficiency (DL%) was calculated as following:

$$\mathrm{DL}\%\;\;=\;\;\frac{\mathrm{Weight}\;\mathrm{of}\;\mathrm{VP}16\;\mathrm{in}\;{\mathrm{SiO}}_{2}@\mathrm{LDH}-\mathrm{VP}16}{\mathrm{Weight}\;\mathrm{of}\;{\mathrm{SiO}}_{2}@\mathrm{LDH}-\mathrm{VP}16}\;\;\times \;\;100\%$$

*Characterizations—FTIR Spectral and Size Distribution by Intensity Study*: FTIR of VP16, SiO_2_@LDH, and SiO_2_@LDH‐VP16 was obtained on a Bruker Vector 22 (Bruker Corporation, Billerica, MA, USA) spectrophotometer in the range of 500–4000 cm^−1^ using the standard KBr disk method (sample: KBr = 1:100).

Size distribution by intensity are determined using DLS at 25 °C by photon correlation spectroscopy (Zetasizer Nano ZS, Malvern Instruments, Malvern, UK), the SiO_2_@LDH‐VP16 (1 mg mL^−1^) are prepared following the dilution that 100 μL samples diluted in 1 mL aqueous solution.

*Characterizations—In Vitro Drug Release*: The in vitro release of SiO_2_@LDH‐VP16 was studied using the dialysis membrane method. In detail, SiO_2_@LDH‐VP16 (20 mg) was dispersed in PBS solution (12 mL) was transferred to a dialysis bag (cutoff size 14 kDa). The bag was dipped into PBS (200 mL) at certain pH (5.8 and 7.4) at 37 °C in a shaking water bath (100 rpm). The VP16 concentrations in the released samples were analyzed by UV‐vis spectroscopy at certain time points.

*Cell Culture and In Vitro Analysis—Cell Culture*: Human pulmonary adenocarcinoma A549 cells are a type of non‐small cell lung cancer cell that was used as a common in vitro model for metastasis. A549 cell line was purchased from the Chinese Academy of Sciences and maintained in RPMI‐1640 with 10% fetal bovine serum (FBS) and 1% penicillin‐streptomycin (Gibco, BRL, Grand Island, NY, USA).

*Cell Culture and In Vitro Analysis—Cellular Cytotoxicity*: A549 cells were seeded into a 96‐well plate at a density of 1 × 10^4^ cells per well and incubated overnight (5% CO_2_, 37 °C). Then, different groups of cells were established as incubated with VP16, LDH‐VP16, SiO_2_@LDH‐VP16, and SiO_2_@LDH at various concentrations (2.5, 5, 10, 20, and 40 μg mL^−1^) for 24 and 48 h. The control group was set up treated with regular medium. Afterwards, 3‐(4,5‐Dimethylthiazol‐2‐yl)‐2,5‐diphenyltetrazolium bromide (MTT) solution (10 μL, 5 mg mL^−1^) was added to the wells. After incubated for another 4 h at 37 °C, the cells were treated with dimethyl sulfoxide (150 μL, DMSO). The absorbance was measured at 490 nm using the microplate reader (ELx800, BioTek Instruments, Inc., Winooski, VT, USA) and cell viability was determined by the following equation:

$$\mathrm{Cell}\;\;\mathrm{viability}\;(\%)\;\;=\;\;\frac{\mathrm{OD}490\left(\mathrm{test}\right)-\mathrm{OD}490\left(\mathrm{blank}\right)}{\mathrm{OD}490\left(\mathrm{control}\right)-\mathrm{OD}490\left(\mathrm{blank}\right)}\;\;\times \;\;100\%$$

*Cell Culture and In Vitro Analysis—Wound Healing Assay*: Wound healing assay was performed as following: A549 cells were seeded in 6‐well plates and incubated overnight to form monolayer cells. Next, the cells were divided into two groups treated with SiO_2_@LDH, VP16, LDH‐VP16, and SiO_2_@LDH‐VP16 in the two concentrations (2.5 and 5 μg mL^−1^), respectively. After treated for 24 h, the cells were washed with PBS and incubated with serum‐starvation medium for 2 h. Then, a wound line on the monolayer was achieved using a micropipette tip. The cells were washed with PBS and incubated for another 6 h. At last, wound healing was checked using the microscope (Nikon, Tokyo, Japan) at 40× magnification and six areas were picked up in each group to quantify cell migration. After another 18 h of incubation, the wound was again assessed.

*Cell Culture and In Vitro Analysis—Transwell Migration Assay*: A549 cells were pre‐cultured with serum‐free media for 4 h and then suspended at density of 1 × 10^5^ per well in serum‐free media (200 μL) containing VP16, LDH‐VP16, SiO_2_@LDH, or SiO_2_@LDH‐VP16 at two concentrations (2.5 and 5 μg mL^−1^), the suspended cells were seeded to upper chamber of 24‐well transwell plates (BD Biosciences) with filters with an 8.0 μm pore size (Costar). At the same time, RPMI‐1640 (600 μL) with FBS (10%) was added to the lower chamber. After 24 h, the cells on the upper surface of the filters were wiped gently using a cotton swab. The migration cells on the low surface were fixed with 95% ethanol, stained with AM‐calcein (Sinopharm Chemical Regent Co, Ltd, Shanghai, China) and then observed under the microscope at 200× magnification.

*Cell Culture and In Vitro Analysis—Transwell Invasion Assay*: Protocol of transwell invasion assay is basically as same as transwell migration assay except one step: before seeding cells, the MatrigelTM Matrix (BD Biosciences) was diluted in serum‐free medium to cover the transwell inserts in the plates.

*In Vitro and In Vivo Angiogenes is Assays—Matrigel Tube Formation Assay*: Tube formation was determined as a classical in vitro angiogenesis assay following reported protocol.^56^ The starving human umbilical vein endothelial cells were seeded onto Matrigel‐coated 96‐well at a concentration of 1 × 10^4^ per well and cultured for 24 h in media (5% FBS, 10 ng mL^−1^ VEGF) with VP16, LDH, SiO_2_@LDH, LDH‐VP16, or SiO_2_@LDH‐VP16 (5 μg mL^−1^). The degree of network formation was quantified using an Image analyzer.

*In Vitro and In Vivo Angiogenesis Assays—Chorioallantoic Membrane (CAM) Assay*: The chick embryos assay was performed as a kind of favored in vivo study as previously described.^57^ Briefly, fertilized chicken eggs were incubated for seven days in the incubator. A round window was opened in the shell and the membrane was detached carefully. The eggs were divided into different groups treated with VP16, LDH, SiO_2_@LDH, LDH‐VP16, or SiO_2_@LDH‐VP16 (5 μg mL^−1^) for a week in the incubator. CAMs were photographed and the level of angiogenesis was estimated using the KS400 imaging assay system.

*In Vitro and In Vivo Angiogenesis Assays—In Vivo Matrigel Plug Assay*: Matrigel assay is another method that is widely used to evaluate neovascularization. The steps were according to established protocols, 600 mL Matrigel containing VEGF (500 ng mL^−1^) was mixed with VP16, LDH, SiO_2_@LDH, LDH‐VP16, or SiO_2_@LDH‐VP16 (5 μg mL^−1^).^58^ The mixture was injected subcutaneously into the flank of 6 weeks old BALB/c mice (Shanghai Laboratory Animal Co. (SLAC), Ltd., Shanghai, China). Negative and positive controls were obtained by giving Matrigel in the absence or presence of VEGF. After 10 d, the plugs were removed and photographed, then fixed in formalin and stained with H&E. The hemoglobin content was determined according to Drabkin's method versus the negative controls. Vessel count was calculated using ImageJ.

*In Vivo Metastasis Assay*: Female athymic nude mice (5–6 weeks old, SLAC Ltd., Shanghai, China) were hosted in SPF room of the animal house in Tongji University and treated following the protocol approved by the Institutional Animal Care and Use Committee at Shanghai Institute of Materia Medica, Chinese Academy of Sciences. Mice were injected into the tail vein with 1 × 10^5^ A549‐Luc cells/100 μL.^59^ To monitor the effect of SiO_2_@LDH‐VP16 against metastasis, mice were treated every each day with 10 mg kg^−1^ VP16, LDH, SiO_2_@LDH, LDH‐VP16, or SiO_2_@LDH‐VP16 though i.p. (5 mice per group). The control group was given the same volume of PBS. Mice were anesthetized with isoflurane and given a single i.p. dose of d‐luciferin in PBS (10 mg mL^−1^, 200 μL). About 5 min after injection, mice were imaged using the IVIS Imaging System (Xenogen, USA) at day 14 and day 44. On day 44, all the mice were euthanized by CO_2_ according to humanitarianism. Lung tissues were collected, fixed in formalin and stained with H&E. The analysis was performed by Living Image 2.5 software (Xenogen). Every other day, the life‐or‐death situation was checked and record to monitor the survive proportions.

*Cellular Uptake Study*: Flow cytometry was used to determine the cellular uptake. A549 cells were seeded to 6‐well plate and incubated with VP16‐FITC, LDH‐VP16‐FITC, or SiO_2_@LDH‐VP16‐FITC (5 μg mL^−1^). After 24 h, cells were re‐suspended in PBS (500 μL) in flow cell tubes and examined above 530 nm by FACS‐calibur flow cytometry (Becton Dickinson, San Jose, CA).

*Co‐Localization Assay*: To further explore the cellular uptake of SiO_2_@LDH‐VP16 nanoparticles, traditional markers were utilized for co‐localization. Dil (red) is a kind of lipophilic fluorescent stains for labeling cell membranes. Tubulin‐Tracker Red is a used as a well‐characterized microtubule marker. While DAPI (blue) was used to marked the cell nucleus.^60^, ^61^ A549 cells were incubated with VP16‐FITC, LDH‐VP16‐FITC, or SiO_2_@LDH‐VP16‐FITC (10 μg mL^−1^, green) for 24 h, washed twice with PBS and then stained with Dil, Tubulin or DAPI for a certain time according to the description of kits (KeyGen BioTECH, Nanjing, China). All the groups were imaged under the confocal microscope (Leica TCS SP5, Leica Microsystems GmbH, Germany).

*Western Blot Assay*: A549 cells were seeded onto a 6‐well plate and treated with VP16, LDH‐VP16, SiO_2_@LDH‐VP16, LDH, or SiO_2_@LDH at a two concentrations (5 and 10 μg mL^−1^). Afterward, treated and untreated cells were harvested after 24 h and 20 μg of protein cellular lysate were fractionated by sodium dodecyl sulfate gel electrophoresis and transferred to the polyvinylidene difluoride (PVDF) membrane. Membranes were probed for ß‐actin, phospho‐ß‐catenin (Ser33/37/Thr41), PI3 Kinase p110α (PI3K), phospho‐mTOR (Ser2448), phospho‐AKT (Ser 473, phospho‐VEGFR‐2, FAK (D2R2E), and Paxillin (D9G12) using specific antibodies (Cell Signaling Technology, Danvers, MA, USA). Protein signals were detected using a enhanced chemiluminescence (ECL) detection system (Santa Cruz Biotechnology, Santa Cruz, CA, USA) and quantified using BandScan 5.0 software.

*Statistical Analyses*: The statistical significance of the differences between the groups was determined using Student's *t*‐test, and one‐way analysis of variance was used for the experiments with multiple groups. For all statistical analyses, a *P* < 0.05 was considered significant.

## Supporting information

As a service to our authors and readers, this journal provides supporting information supplied by the authors. Such materials are peer reviewed and may be re‐organized for online delivery, but are not copy‐edited or typeset. Technical support issues arising from supporting information (other than missing files) should be addressed to the authors.

Supplementary
