# Supplementary material for: Anti‐Metastatic and Anti‐Angiogenic Activities of Core–Shell SiO2@LDH Loaded with Etoposide in Non‐Small Cell Lung Cancer
Source: Adv Sci (Weinh). 2016 Oct 8;3(11):1600229. doi: 10.1002/advs.201600229 (PMC5102674; doi:10.1002/advs.201600229)
Supplement: Supplementary file 1 — Supplementary [file ADVS-3-0s-s001.pdf]

Copyright WILEY-VCH Verlag GmbH & Co. KGaA, 69469 Weinheim, Germany, 2016.

Supporting Information

for *Adv. Sci.,* DOI: 10.1002/advs.201600229

Anti-Metastatic and Anti-Angiogenic Activities of Core–Shell SiO_2@LDH Loaded with Etoposide in Non-Small Cell Lung Cancer

Yanjing Zhu, Rongrong Zhu, Mei Wang, Bin Wu, Xiaolie He, Yechang Qian,* and Shilong Wang**

Supporting Information

Anti-metastatic and Anti-angiogenic activities of Core–shell SiO_2@LDH loaded with Etoposide in Non-small Cell Lung Cancer

Yanjing Zhu[1#], Rongrong Zhu[1#] *, Mei Wang[1], Bin Wu[1], Xiaolie He[1], Yechang Qian[2]*, Shilong Wang[1]*

[1] Research Center for Translational Medicine at East Hospital, School of Life Science and Technology, Tongji University, Shanghai, China, 200092.

[2] Department of Respiratory Disease, Baoshan District Hospital of Integrated Traditional Chinese and Western Medicine, Shanghai, China, 201900.

[#] Both authors contributed equally to this work

* Corresponding Author

Yechang Qian

Tel +86 (021) 65918108 Fax +86 (021) 36070892 Email: qian_yel@163.com

Rongrong Zhu

Tel +86 (021) 65982595 Fax +86 (021) 65982595 Email: rrzhu@tongji.edu.cn

Shilong Wang

Tel +86 (021) 65982595 Fax +86 (021) 65982595 Email: wsl@tongji.edu.cn

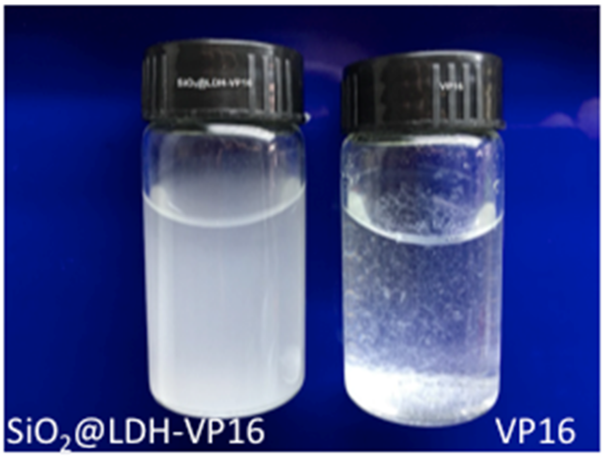

Figure S1. SiO_2@LDH-VP16 and VP16 were suspended in PBS (20 mg/mL) in room temperature. The photo was taken after standing for 2 h.

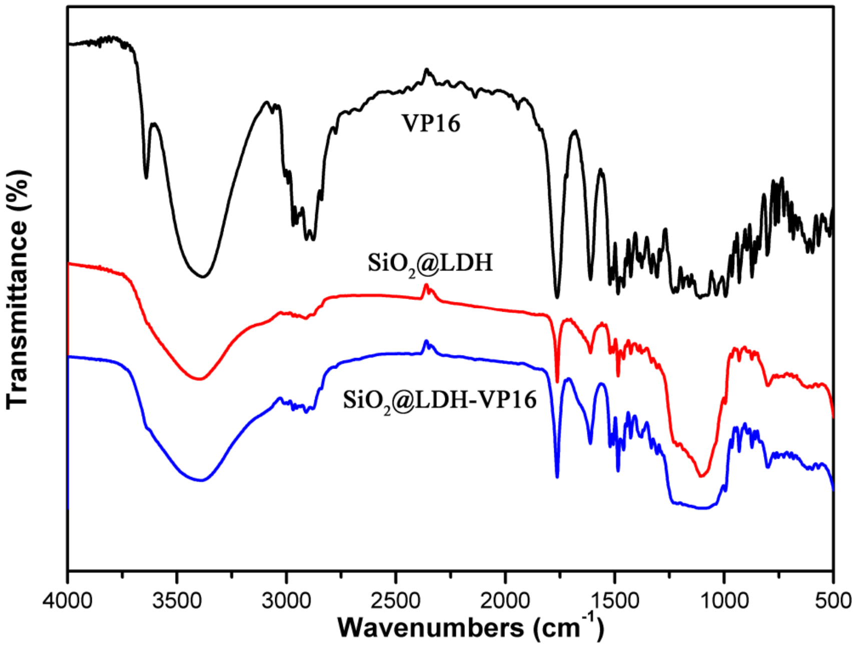

Figure S2. FTIR spectra of VP16, SiO_2@LDH and SiO_2@LDH-VP16.

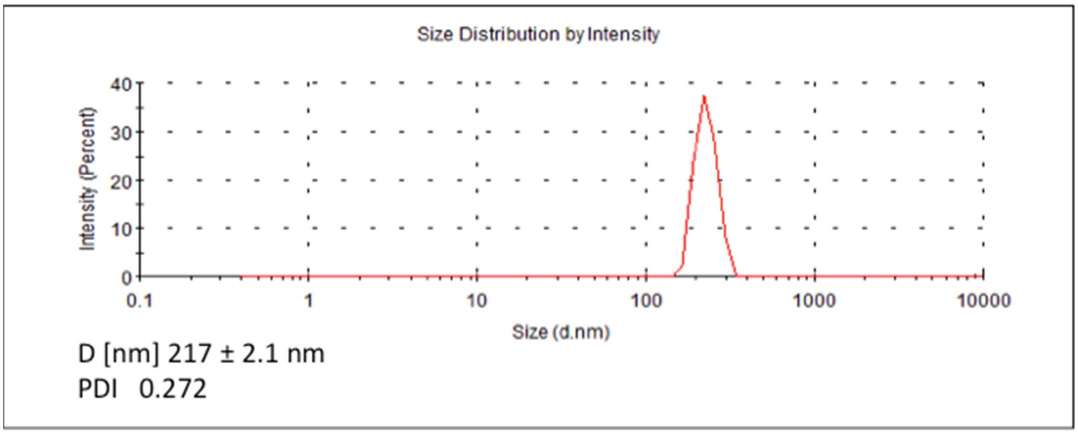

Figure S3. Size Distribution by Intensity of SiO_2@LDH-VP16

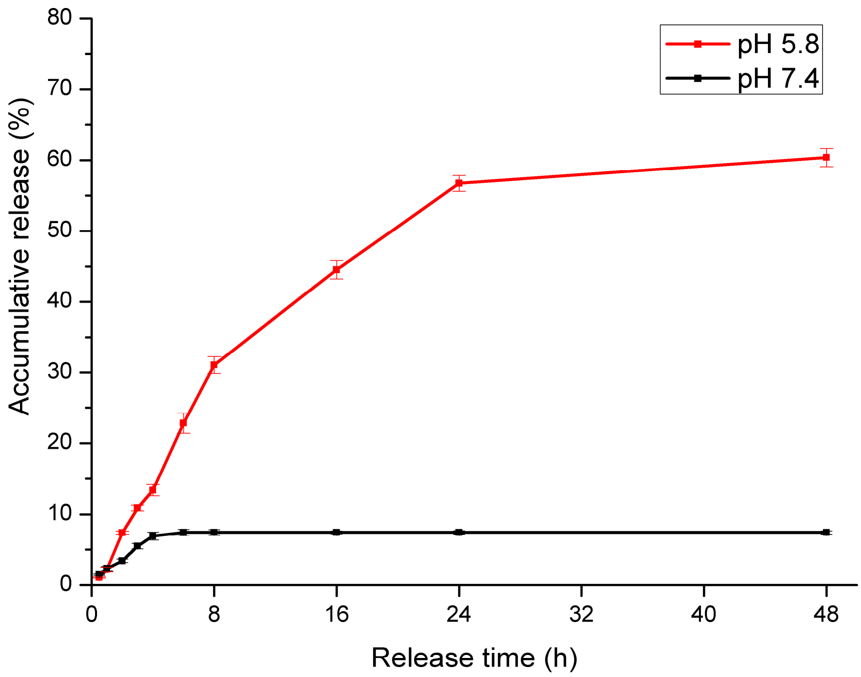

Figure S4. In vitro release profile of VP16-loaded SiO_2@LDH nanoparticles. Drug release study was performed at 37°C under shaking (100 rpm) using a dialysis membrane bag containing phosphate buffered saline (0.01 M, pH 7.4 and pH 5.8) as a sink solution. Results shown as mean ± standard deviation; n=3.
